# Y chromosome in Turner syndrome: review of the literature

**DOI:** 10.1590/S1516-31802009000600010

**Published:** 2010-05-21

**Authors:** Rose Mary Rocco de Oliveira, Ieda Therezinha do Nascimento Verreschi, Monica Vannucci Nunes Lipay, Lilian Piñero Eça, Alexis Dourado Guedes, Bianca Bianco

**Affiliations:** I BSc. Postgraduate Course on Cell and Molecular Biology, Centro de Extensão Universitária (CEU), São Paulo, Brazil.; II MD, PhD. Associate professor, Division of Endocrinology, Department of Medicine, Universidade Federal de São Paulo (Unifesp); and Professor of Postgraduate Course on Cell and Molecular Biology, Centro de Extensão Universitária (CEU), São Paulo, Brazil.; III PhD. Affiliated professor, Division of Endocrinology, Department of Medicine, Universidade Federal de São Paulo (Unifesp), São Paulo, Brazil.; IV MD, PhD. Postgraduate student, Division of Endocrinology, Department of Medicine, Universidade Federal de São Paulo (Unifesp), São Paulo, Brazil.; V BSc, PhD. Biomedical specialist in the Department of Medicine and postgraduate student, Division of Endocrinology, Department of Medicine, Universidade Federal de São Paulo (Unifesp); and Professor of Postgraduate Course on Cell and Molecular Biology, Centro de Extensão Universitária (CEU), São Paulo, Brazil.

**Keywords:** Turner syndrome, Chromosomes, human, Y, Sex cord-gonadal stromal tumors, Genes, sry, Mosaicism, Síndrome de Turner, Cromossomos humanos Y, Tumores do estroma gonadal e dos cordões sexuais, Genes sry, Mosaicismo

## Abstract

Turner syndrome (TS) is one of the most common types of aneuploidy among humans, and is present in 1:2000 newborns with female phenotype. Cytogenetically, the syndrome is characterized by sex chromosome monosomy (45,X), which is present in 50-60% of the cases. The other cases present mosaicism, with a 45,X cell line accompanied by one or more other cell lines with a complete or structurally abnormal X or Y chromosome. The presence of Y-chromosome material in patients with dysgenetic gonads increases the risk of gonadal tumors, especially gonadoblastoma. The greatest concern is the high risk of developing gonadoblastoma or other tumors and virilization during puberty if chromosome Y-specific sequences are present. The role of the Y chromosome in human oncogenesis is still controversial. Even though gonadoblastoma is a benign tumor, it can undergo transformation into invasive dysgerminoma in 60% of the cases, and also into other, malignant forms of germ cell tumors. Although some authors have questioned the high incidence of gonadoblastoma (around 30%), the risk of developing any kind of gonadal lesion, whether tumoral or not, justifies investigation of Y-chromosome sequences by means of the polymerase chain reaction (PCR), a highly sensitive, low-cost and easy-to-perform technique. In conclusion, mosaicism of both the X and the Y chromosome is a common finding in TS, and detection of Y-chromosome-specific sequences in patients, regardless of their karyotype, is necessary in order to prevent the development of gonadal lesions.

## RESUMO

A síndrome de Turner (ST) é uma das aneuploidias mais comuns em humanos e está presente em 1:2000 recém-nascidas com fenótipo feminino. Citogeneticamente, a síndrome é caracterizada por uma monossomia de cromossomo sexual (45,X) em 50-60% dos casos. Os demais casos apresentam mosaicismo com uma linhagem celular 45,X acompanhada de outra(s) com o cromossomo X ou Y íntegros ou com alterações estruturais. A presença de material do cromossomo Y em pacientes com gônadas disgenéticas aumenta o risco de tumores gonadais, especialmente gonadoblastoma. A consideração mais importante diz respeito ao elevado risco de desenvolvimento de gonadoblastoma ou outros tumores e a virilização na puberdade se sequências cromossomo Y-específicas estiverem presentes. O papel do cromossomo Y na oncogênese dos cânceres humanos ainda é controverso. Apesar de o gonadoblastoma ser um tumor benigno, ele pode transformar-se num disgerminoma invasivo em 60% dos casos e também em outras formas malignas de tumores de células germinativas. Apesar de alguns autores questionarem a alta incidência (em torno de 30%) de gonadoblastoma, o risco do desenvolvimento de qualquer tipo de lesão gonadal, tumoral ou não, justifica a pesquisa de sequências do cromossomo Y por PCR (reação de polimerase em cadeia), técnica de alta sensibilidade, baixo custo e fácil execução. Em conclusão, o mosaicismo cromossômico tanto do X como do Y é um fato comum na ST e a detecção de sequências cromossomo Y-específicas nas portadoras, independentemente do seu cariótipo, é necessária para prevenir o desenvolvimento de lesões gonadais.

## INTRODUCTION

In 1938, Henry Turner^1^ described a group of female adolescents with primary amenorrhea, sexual infantilism and short stature. The patients also presented *cubitus valgus*, webbed neck, widely spaced nipples, low posterior hairline and lymphedema of hands and feet. Four years later, Varney et al.^2^ and Albright et al.,^3^ independently from each other, studied patients with clinical signs resembling those described by Turner and demonstrated that, after puberty, they presented a high level of urinary gonadotropins, thus establishing that there was an abnormality of gonadal function rather than a hypothalamic or hypophyseal deficiency. In l944, Wilkins and Fleischmann^4^ performed histological analyses and observed that patients with the clinical signs described by Turner,^1^ Varney et al.^2^ and Albright et al.^3^ probably had streak gonads, and that all those authors were studying the same syndrome: Turner’s syndrome.

**Figure 1.**
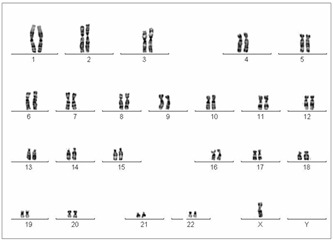
Turner syndrome karyotype (45,X).

Turner’s syndrome (TS) is one of the most common types of aneuploidy in humans, and is present in 1:2000 newborns with female phenotype.^5^^,^^6^ The first karyotype investigation in a patient with TS was performed in England, in 1959, by Ford et al.^7^ These authors were able to describe X-chromosome monosomy, i.e. the 45,X karyotype, which is the type most frequently found among patients with TS^8^ (Figure 1).

## CLINICAL CHARACTERISTICS

Individuals with TS present extremely variable phenotypes. Despite their undifferentiated gonads, they have clearly female external and internal genitals. However, their uterus is small, since its full development depends on hormonal stimuli. The gonads are usually two fibrous streaks in which no germ cells or follicular formations, but only tissue resembling the stroma of the ovarian cortex is detected.^9^^,^^10^

**Figure 2.**
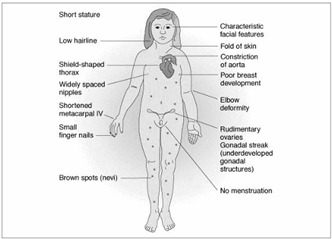
Clinical-phenotypic findings in Turner.^11^

Other signs that are found may include dysmorphic auricles with the longitudinal axis in an oblique position, eyelids with antimongoloid slanting, internal epicanthal folds, high-arched palate, retrognathism, widely spaced nipples, short sternum, cardiovascular malformations (the most common are coarctation of the aorta and ventricular septum defects), renal malformations (such as horseshoe kidneys, urethral duplication and unilateral kidney agenesis) and hypoplasia of the fourth or fifth metacarpal and metatarsal bones. Breast growth in TS patients is much slower than in normal individuals. Since women with TS present gonadal dysgenesis, the endocrine changes that are typical of puberty do not occur, and reports of primary amenorrhea are frequent^9^^,^^10^ (Figure 2).^11^

In 1997, Rao et al.^12^ isolated a gene named *SHOX* (short stature homeobox gene) that is located at Xp22 and Yp11.3, in the pseudoautosomal region of the sex chromosomes. The haploinsufficiency of this gene is believed to be responsible for the short stature and for several skeletal anomalies presented by TS patients.

TS patients present high frequencies of autoimmune diseases, particularly hypothyroidism, celiac disease and diabetes mellitus, which so far remain unexplained.^5^^,^^10^^,^^13^ Moreover, some authors have suggested that there is a higher risk of autoimmune diabetes among TS patients with karyotypes presenting an X isochromosome.^4^ An isochromosome of the long arm of the X chromosome (Xq) results from deletion of the short arm and duplication of the long arm of one of the X chromosomes, and this is the most common structural abnormality found in TS cases.

## GENETIC CHARACTERISTICS

Cytogenetically, the Turner syndrome is characterized by sex chromosome monosomy (45,X) in phenotypically female individuals. This karyotype is found in 50-60% of the cases.^5^^,^^14^ The other cases are mosaics with a 45,X cell line accompanied by others with two or more X chromosomes or with structural anomalies. Such structural anomalies of the X chromosome (isochromosomes of the long arm, dicentric chromosomes, deletion of the short arm or ring chromosomes) are present in approximately 30% of the cases, in homogeneous karyotypes or in mosaics that include a 45,X cell line. Finally, around 5% are accounted for by patients with structural abnormalities of the Y chromosome (isochromosomes of the long arm and dicentric chromosomes) and mosaics that include a cell line accompanied by others with at least one Y chromosome, whether complete or not.^5^^,^^8^

Data from the literature show that a second sex chromosome is necessary for the fetus to survive, and therefore virtually every liveborn 45,X individual should present more than one cell karyotype line, thus constituting a mosaic.^15^ This condition would be necessary for at least some organs, during a certain period of embryogenesis. This hypothesis is based on two main points: the frequency of sex chromosome mosaicism is much higher in liveborn infants with TS than in aborted fetuses; and an estimate that approximately 99% of the embryos with a pure 45,X karyotype die in utero.^16^^,^^17^

Furthermore, comparison with other numerical chromosomal anomalies, such as Down syndrome or chromosome 18 trisomy, reveals that the incidence of TS does not increase with maternal age.^18^^,^^19^ These findings argue against meiotic nondisjunction as the main mechanism for the generation of a 45,X karyotype. Clinically, in contrast to other chromosomal syndromes, even patients with a pure 45,X karyotype present completely distinct phenotypes. Except for short stature, which seems to be a general characteristic, all other findings are inconsistent. One of the possible explanations for this fact might be undetected mosaicism, since the diagnosis is usually made by analyzing between 5 and 30 peripheral blood lymphocytes to determine the karyotype,^20^ and the second cell line is often present as a proportion of no more than 1 to 2% of the individual’s cells.

In around 99% of human conceptuses with a 45,X karyotype, natural loss occurs during the first stage of embryonic development.^15^^,^^17^ Only 1% of these fertilizations are successful, and they generally display TS characteristics.^1^ Both embryonic mortality and the characteristic TS phenotype are considered to result from monosomy of genes that are common to the X and Y chromosomes (pseudoautosomal region).^21^ In women, it is clear that these genes are expressed both in the active and in the inactivated X chromosome, as a means of ensuring the proper amount of gene product. It is believed that one or more of these genes are responsible for TS.^22^^,^^23^

## DETECTION OF MOSAICISM

The detection of mosaicism is determined mainly by four factors: the type and number of tissues analyzed; the number of cells studied;^16^ the sensitivity of the techniques used; and the possibility of selection, which can result in disappearance of cell lines.^15^^,^^24^ A small percentage of mosaicism cannot be detected by the classical cytogenetic technique, because this kind of analysis requires a large number of cells. The use of molecular techniques such as fluorescence *in situ* hybridization (FISH) and the polymerase chain reaction (PCR) substantially improves the detection of low-frequency cell lines and possible structural abnormalities.^23^^,^^25^

Peripheral blood lymphocytes are generally the material of choice for cytogenetic analysis on patients suspected of having TS, since this tissue is easy to obtain. In routine laboratory diagnosis, the classical cytogenetic analysis is performed in thirty metaphases, which allows detection of 10% mosaicism. If the hypothesis that all patients with Turner syndrome carry mosaicism is true, it is possible that cell lines that are present in blood at frequencies below 10% go undetected by this method.^26^

Another issue to be considered is the analysis of different tissues, since mosaicism may not be detected in peripheral blood, but may be significant in tissue samples of different embryonic origin, for example oral mucosa.^26^^,^^27^^,^^28^^,^^29^

The presence of Y-chromosome material in individuals with TS can be investigated both cytogenetically and by using various molecular approaches.^30^ The latter presents the advantages of not needing cell cultures and requiring only a rather small amount of material. Thus, the use of molecular methods for identifying Y-chromosome-specific sequences has become an indispensable tool for detecting hidden mosaicism.^31^^,^^32^^,^^33^^,^^34^

Nazarenko et al.^35^ also observed that cytogenetic analysis may not provide precise information on the presence of chromosomal mosaicism in patients with TS. Additional analysis of cells from tissues of different embryonic origins (mesodermal lymphocytes and ectodermal oral epithelium cells, for instance) allowed greater precision in defining the cytogenetic diagnosis. FISH analysis with a probe for the X chromosome allowed a more accurate result, thus showing that 29% out of fifty patients with pure X monosomy detected by ordinary karyotyping actually presented mosaics. In addition, according to these authors, distinct chromosomal constitution was observed in different tissues from the same individual, thereby highlighting the possibility that chromosomal abnormalities or the presence of a second sex chromosome may be absent in blood but present in other tissues.

According to Mendes et al.,^36^ mosaicism is present in 25% of TS karyotypes. Y-chromosome-specific sequences can be observed in approximately 40% of these cases. According to López et al.,^32^ the difficulty in comparing the frequency of Y-chromosome-specific sequences or of the Y chromosome itself in patients with TS is due mainly to the variability in the number of patients analyzed; the frequency of mosaicism with a normal and an abnormal X chromosome; the number of cases with marker chromosomes; the molecular methodology applied in each study; and the Y-chromosome-specific sequences studied. These authors used a combination of classical cytogenetics and molecular deoxyribonucleic acid (DNA) analysis methods to study the presence of Y-chromosome-derived material in 50 patients with Turner syndrome. Their molecular results revealed that the frequency of Y-chromosome-specific sequences was 12%, whereas it had previously been found to be 2%, using classical cytogenetics.

Chu et al.^33^ stated that PCR is more effective than cytogenetic analysis for detecting hidden mosaicism involving the Y chromosome, and may be even more effective using a multiple-tissue approach, thereby raising the chances of revealing mosaicism, if present.

Guedes et al.^37^ studied a girl who, despite her 45,X/46,X,der(Y) karyotype, displayed no signs of virilization and/or clinical features of TS, except for decreased growth speed. After prophylactic gonadectomy, in view of the risk of developing gonadoblastoma, samples from blood and gonad material were studied by means of FISH and PCR, to search for Y-chromosome-specific sequences. These analyses revealed that the Y-derived chromosome was actually a Yp isodicentric chromosome and that there was a significant difference in the distribution of the mosaicism between the two tissues studied. Although 97.5% of the cells analyzed in peripheral blood were 46,X,idic(Yp) with duplication of the *SRY* gene, this did not determine any degree of male sex differentiation, since 60% of the cells analyzed in the gonadal tissue were 45,X, thus suggesting that in this patient, the tissue-specific mosaicism contributed towards female sex development.

## Y CHROMOSOME AND RISK OF GONADAL TUMOR DEVELOPMENT

Abnormalities in gonad organogenesis can lead to the development of gonadal tumors,^38^ especially in patients with dysgenetic gonads.^39^ Patients with disorders of sexual development are at increased risk of developing tumors originating from germ lines, also known as germ-cell tumors.^40^ Several risk factors have been identified for these kinds of germ-cell tumors, particularly those relating to gonads, including cryptorchidism and gonadal dysgenesis.^40^^,^^41^^,^^42^

The precursor lesion for dysgenetic gonad tumors is named gonadoblastoma.^41^ This has the potential to progress towards invasive germ-cell tumors, particularly dysgerminoma, and, less frequently, towards components of other tumors, such as embryonic carcinoma, teratoma, yolk sac tumor and choriocarcinoma.^43^

Gonadoblastoma is a mixed tumor of undifferentiated cells that recapitulates gonadal development^41^ and is able to originate dysgerminoma in 60% of the cases.^44^ Hyperandrogenism is a phenomenon commonly associated with gonadoblastoma, especially in cases of coexistence with dysgerminoma.^44^

There is strong evidence that gonadoblastoma results from a disorder in germ cell maturation. This model is supported by epidemiological and morphological observations, such as the presence of immunohistochemical germ cell markers (placental alkaline phosphatase) and proto-oncogenes (*c-KIT*).^45^^,^^46^^,^^47^

The genes *SRY*^16^^,^^20^^,^^32^^,^^36^^,^^48^^,^^49^^,^^50^^,^^51^^,^^52^^,^^53^^,^^54^^,^^55^^,^^56^ and *DYZ3*^16^^,^^32^^,^^34^^,^^48^^,^^49^^,^^50^^,^^51^^,^^53^^,^^55^^,^^56^^,^^57^ are the sequences most commonly used in studies. Controversy still exists regarding which Y-chromosome markers are the most relevant. In general, the *SRY* gene is the sequence most used, because of its location and important role in the sex differentiation cascade.^58^

However, with the identification of novel genes on the Y chromosome and the so-far unconfirmed suspicion that there is a specific gene associated with the development of gonadoblastoma, other regions have been associated with the development of this tumor.^59^

*GBY* (gonadoblastoma locus on the Y chromosome) is a gene that is assumed to be related to the presence of tumors originating from dysgenetic gonads. This gene is probably located within a small region of the short arm of the Y chromosome, close to the centromere.^59^*TSPY* (testis-specific protein Y-encoded) is a candidate gene for the *GBY* locus, which is possibly related to the development of gonadoblastoma and the involvement of a specific region of the Y chromosome.^60^^,^^61^ Hildenbrand et al.^62^ studied a patient with TS and a 45,X/46,X,+mar karyotype who developed unilateral gonadoblastoma. Cytogenetic and molecular studies confirmed that the marker was derived from the Y chromosome. These authors investigated the gonad material by immunohistochemistry for expression of the *TSPY* gene, and the results revealed a high level of TSPY protein expression.

Furthermore, the *POU5F1* (*OCT4*) gene*,* located at 6p21.31, is expressed in pluripotent stem cells and germ cells in mice and humans.^63^^,^^64^^,^^65^^,^^66^^,^^67^^,^^68^ Extensive immunohistochemical screening for POU5F1 protein expression has been carried out on several types of germ cell tumors using microarrays and has shown that their immunoreactivity is only detectable in gonadoblastoma, seminoma, dysgerminoma and embryonic carcinoma cells.^43^

Considering that detection of Y-chromosome-specific sequences in patients with Turner syndrome is necessary in order to prevent the development of gonadoblastoma,^69^ clinical characteristics such as signs of hyperandrogenism should also be considered in deciding on the therapeutic approach to be adopted for such patients. This is important because the administration of growth hormone (somatotropin) to patients carrying Y-chromosome fragments may lead to the development of this neoplasm or other androgen-secreting tumors,^70^ although this is still a controversial matter. Treatment with growth hormone is indicated for TS patients and the results have been satisfactory, but the long-term effects resulting from this treatment are still under observation.^71^

The *SRY* gene, whose major role in sex determination and differentiation has been well known ever since the first studies of Page,^60^ represents an intermediate link in the signaling chain that occurs during embryonic development. It serves as an activator and is also regulated by several genes (*SOX-9*; *WT-1*; *SF-1*, etc.). This gene is of fundamental importance in cell differentiation and, consequently, in determining the gonadal microenvironment. The interaction starts in the presence of androgens.

It has traditionally been recommended that a search for Y-chromosome fragments in TS should only be performed under two circumstances: when there are signs of virilization and/or when there is a marker chromosome not identified by classical cytogenetics.^72^^,^^73^ Nevertheless, when Canto et al.^74^ used PCR to study 107 Turner syndrome patients with a 45,X karyotype, they identified Y-chromosome material in ten (9.3%) of them. Prophylactic gonadectomy was indicated, and two of the six patients who agreed to undergo the surgery presented gonadoblastoma, thus indicating an incidence of 33%.

Similarly, Bianco et al.^34^ studied different tissue samples from 20 TS patients by means of PCR and found that seven (35%) of the 45,X patients presented Y-chromosome-specific sequences in at least one of the tissues studied. Four (14%) of these patients underwent prophylactic gonadectomy, and bilateral gonadoblastoma was found in a 16-year-old girl. In this case, the presence of Y-chromosome sequences was not associated with virilization, thus reinforcing the idea that absence of this characteristic does not rule out the possibility of the presence of hidden Y chromosome fragments.

In addition, Bianco et al.^55^ investigated the presence of Y-chromosome mosaicism (*SRY*, *TSPY* and *DYZE*) in 87 TS patients by means of PCR, along with its association with the development of gonadal tumors and/or nontumoral androgen-producing lesions. The data revealed hidden Y-chromosome mosaicism in 18.5% of the patients. The *SRY* sequence was detected in all of these patients, while 4.6% of them presented the *DYZ3* repeat region and 4.6% of them presented the *TSPY* gene. Eleven of the patients with Y-positive sequences agreed to undergo prophylactic surgery. In two cases, bilateral gonadoblastoma was found and, in another case, histopathological analysis on the gonads revealed hilus cell hyperplasia. In a further case, hilus cell hyperplasia and stromal luteoma were found. These authors concluded that a systematic search for hidden Y-chromosome mosaicism, especially for the *SRY* gene, in Turner syndrome patients, was justified because of the possibility of preventing gonadal lesions.

## CONCLUSION

The role of the Y chromosome in human oncogenesis is still controversial. However, identification of Y-chromosome mosaicism is clinically important because of the high risk that tumors such as gonadoblastoma or other, nontumoral androgen-producing lesions might develop in the dysgenetic gonads of patients with hidden Y-chromosome mosaicism or Y-chromosome-specific sequences. Although gonadoblastoma is a benign tumor, it can undergo transformation into invasive dysgerminoma in 60% of the cases, and can also turn into other malignant forms of germ-cell tumors. Even though some authors have questioned the high incidence of gonadoblastoma (around 30%), the possibility that some kind of gonadal lesion might develop, whether tumoral or not, justifies investigation of Y-chromosome-specific sequences by means of PCR, which is a highly sensitive, low cost and easy-to-perform technique.

In conclusion, mosaicism of both the X and the Y chromosome is a common finding in TS, and detection of Y-chromosome-specific sequences in such patients, regardless of their karyotype, is necessary in order to prevent the development of gonadal lesions.
